# A cross-sectional feasibility study of an English-language survey in Switzerland using the Patient-Reported Outcomes Measurement Information System-29

**DOI:** 10.3389/fresc.2026.1840636

**Published:** 2026-07-31

**Authors:** Matthew Kerry, Thanh Elsener

**Affiliations:** 1 Health Department, Zürich University of Applied Science (ZHAW), Winterthur, Switzerland; 2 School of Medicine, Institute of Biomedical Ethics, University of Zürich, Zürich, Switzerland

**Keywords:** English speakers, health survey, PROMIS-29, psychometrics, Switzerland

## Abstract

**Study aims:**

English is the fourth most-used language in Switzerland at 6%, yet no health survey has specifically sampled English-speaking respondents, despite having compulsory health insurance. The purpose of this prospective survey is to explore the feasibility of collecting empirical evidence on the health status of English-speaking respondents living in Switzerland. In addition to reporting psychometrics, we further examine substantive correlates of subjective health, such as migration status, residency duration, and planned residency in Switzerland.

**Methods:**

A cross-sectional design with purposive sampling was used to collect *n* = 156 English-speaking respondents between December 2024 and January 2025. The Patient-Reported Outcomes Measurement Information System (PROMIS-29 v2.1) was administered. Several sociodemographic characteristics (age, sex, education, income, chronic condition, migration background, nationality, and employment) were also collected to inspect the convergent validity of the PROMIS-29 with external correlates. The measurement properties inspected included descriptives (normality and floor/ceiling effects), internal consistency reliability (*ω* ≥ 0.70), structural validity [root-mean-squared error of approximation (RMSEA) < 0.06], and convergent validity (correlation *r* = 0.30–0.50).

**Results:**

*N* = 156 of 215 respondents completed the health survey (73% completion). A chronic condition was reported by 45% of the respondents. Regarding instrumentation, 5/7 scales exhibited normal distributions, and all scales exhibited high reliability (0.83–0.93). Structural validity evidence was mostly supported, but the smaller sample size compromised the overall comparative fit index (0.93). Finally, all scales exhibited moderate-strong convergence validity with self-rated health (*r* = 0.29–0.58).

**Conclusion:**

English-speaking respondent uptake seems to be feasible with a relatively high completion rate of 73%. Furthermore, a preliminary inspection of psychometrics indicated good quality evidence. Future research should expand on questions regarding health service utilization and remaining psychometric criteria, such as criterion validity, responsiveness, and cross-cultural validity with Switzerland’s three primary spoken languages.

## Introduction

Population health surveys are common in Switzerland, with national cross-samples of well-known legacy instruments, such as EQ-5D (1) and the 36-Item Short Form Health Survey (SF-36) (2). Sensibly, these studies are conducted in Switzerland’s three primary national languages, namely, German (62%), French (23%), and Italian (8%) (2). Interestingly, English is reported as the fourth most-used language in Switzerland (6%), but no health surveys have been administered specifically targeting English speakers that we are aware of (3).

The aim of the current study, therefore, is 2-fold: (1) administer a newer health instrument developed in modern psychometrics frameworks entitled the Patient Reported Outcomes Measurement Information System (PROMIS-29) and (2) assess the feasibility, empirical quality, and potential value of collecting English-respondent health surveys in Switzerland. Specifically, we use the COnsensus-based Standards for the selection of health Measurement Instruments (COSMIN) criteria to inspect the PROMIS-29’s structural validity, internal consistency reliability, and convergent validity via inspection of its nomological network with external correlates in expected directions (4).

The PROMIS-29 was selected for examination because it is a newer instrument developed on item response theory (IRT) rather than traditional classical test theory principles (5). Instruments developed on IRT may ensure that only informative or appropriate items are administered. For example, a Swiss-national survey using the SF-36 reported nearly 25% floor/ceiling effects (2). In addition, a recent COSMIN systematic review of generic patient-reported outcome measures in Switzerland reported the EQ-5D as not recommendable for further use, and the SF-36 was found to be potentially useful, but is a longer instrument. Therefore, the PROMIS-29 may be a potentially valuable instrument balancing response burden with health info specificity.

## Materials and methods

The current study was deemed to have exemption status by the University of Zurich Ethics Committee due to its non-interventional design and anonymized data (exemption granted 10 December 2024). Informed consent was provided by all potential participants.

A prospective cross-sectional design with purposive sampling and survey methodology was implemented. Specifically, English-affiliated social and sports clubs and associations were contacted either through membership or through group administrators with an invitation to participate in the health survey. Major cities were targeted based on the probabilistic concentration of English speakers living in Switzerland, e.g., Basel, Geneva, Zug, and Zürich. Administrators replied with agreement to disseminate our study invitations. Eight of 10 organizations replied affirmatively, with one declining and one unavailable for follow-up contact. The study invitation stated the inclusion criteria of adults aged 18–65 years and native or fluent English speakers (>C2).

The PROMIS-29 was administered, comprising 28 five-point Likert-scale items representing seven scales of four items each (Physical Function, Anxiety, Depression, Fatigue, Sleep Disturbance, Social Roles and Activities, and Pain Interference). An additional pain-intensity item was recorded on a 10-point sliding scale (Supplementary File 1).

All analyses were conducted in IBM SPSS v29 and IRTPRO v6. Normally distributed data are expressed as means and standard deviations and non-normally distributed data as medians and interquartile ranges (IQRs). The COSMIN criteria guided our inspection of distributional descriptives (normality and floor/ceiling effects), internal consistency reliability (*ω* ≥ 0.70), structural validity [root-mean-squared error of approximation (RMSEA < 0.06], and convergent validity (correlation *r* = 0.30–0.50) (4). Specifically, normality assumptions, floor/ceiling effects, internal consistency, and correlations were inspected in SPSS. Structural validity via confirmatory factor analysis was inspected in IRTPRO.

## Results

Of *n* = 215 respondents, *n* = 156 completed the survey (73% completion: no missing data). PROMIS-29 missing values by item ranged from 1% (items 2, 3, 10, and 15) to 6% (items 22, 24, and 28). Eight items had no missing data, and 94% answered all PROMIS-29 items. Given the low prevalence of missing user data (6%), Little’s Missing Completely at Random (MCAR) test was conducted and exhibited non-significance [χ^2^(419) = 404.81, *p* = 0.68], permitting multiple imputation of missing user values. Although an additional *n* = 20 were identified as having taken the survey in German, we could not determine if they were non-native English speakers. We retained these respondents in our analyses because we were focused on “sample responsivity” rather than “English measurement validation,” i.e., we did not want to artificially deflate the response rate by removing these respondents. However, we compared comprehensive analyses between both versions of the data, with no substantive changes in statistical significance or sign direction. That is, we conducted a model-moment sensitivity analysis with the full and reduced samples to inspect for distortions to parameter estimates. We found no substantive bias, but the interpretation of results must still be cautious. Summary sample descriptive characteristics are displayed in Table 1.

**Table 1:** Summary of the participants’ characteristics.

Variable	Median [IQR]/(%)
Age (years)	34 [29–45]
Sex (female) (%)	31 (20)
Chronic condition (%)	50 (45)
Physical	35 (22)
Mental	3 (2)
Both	11 (7)
Self-rated health	2 [2–3]
Excellent	23 (15)
Very good	71 (46)
Good	46 (30)
Mediocre	10 (6)
Poor	6 (4)
Income	175,000 [105,000–225,000]
Migration background (yes, %)	115 (74)
Nationality (%)	
Non-response	90 (58)
American/Swiss-American	19 (12)
UK/Swiss-UK	8 (5)
Canadian/Swiss-Canadian	6 (4)
Spanish	3 (2)
Polish	3 (2)
Other	27 (17)
Education level (%)	
Compulsory	1 (0.5)
Vocational training	6 (4)
Secondary level	6 (4)
Tertiary/university level	74 (47)
Post-grad and higher	69 (44)
Employment (%)	
0% or jobless	14 (9)
1–40%	3 (2)
41–60%	7 (5)
61–80%	17 (11)
81–100%	85 (55)
Retired	15 (10)
Housewife/husband	5 (3)
Other (professional training, holiday)	10 (6)
Years spent in Switzerland	6 [3–18]
Years plan to be in Switzerland	3 [1–20]
Do not know	9 (6)
≤2 years	14 (9)
3–10 years	16 (10)
11–20 years	9 (6)
21–45 years	12 (7)
50+ years/indefinitely	53 (34)

### Descriptives and reliability

**Figure 1:**
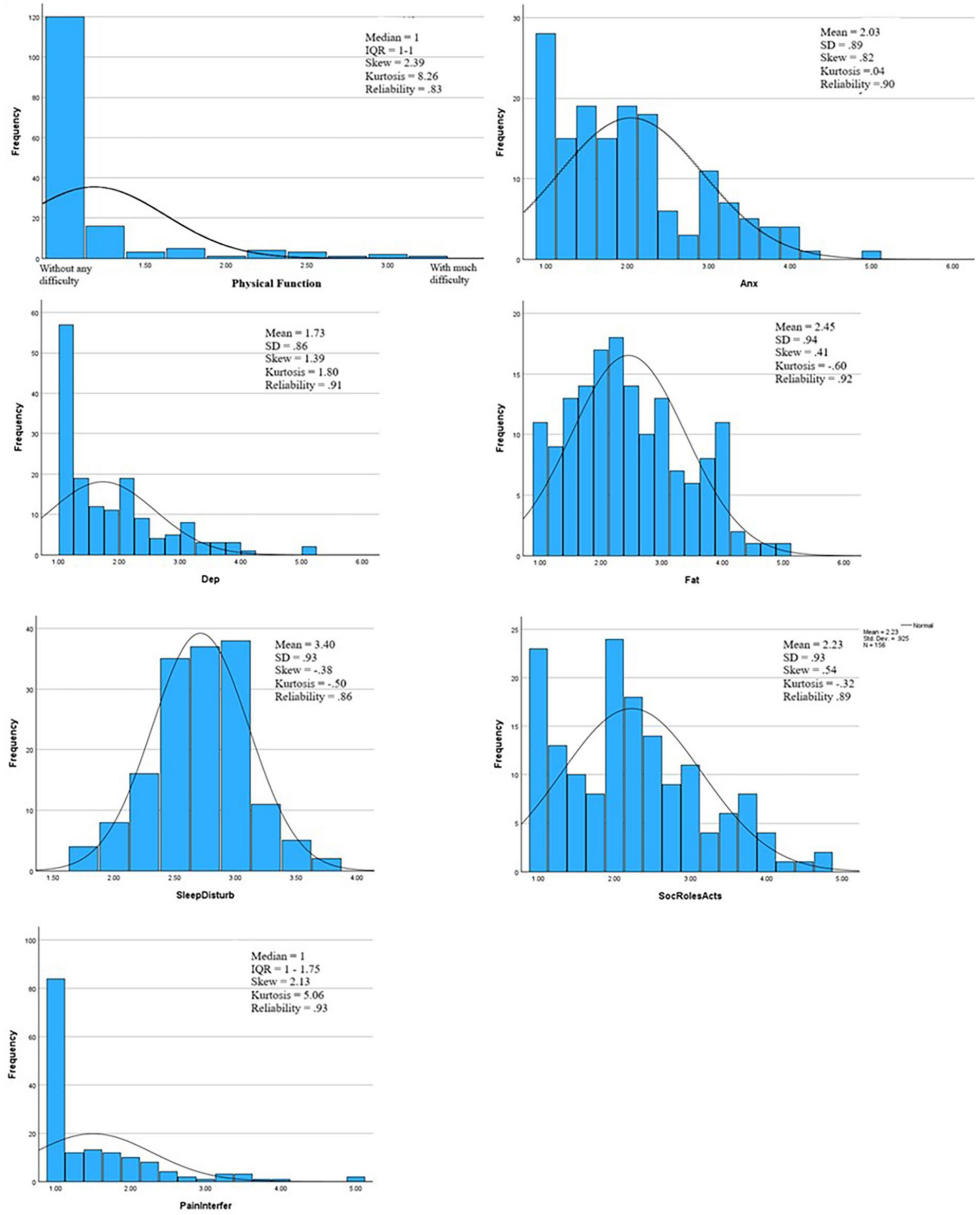
PROMIS-29 domain histograms and univariate descriptive statistics.

Univariate item-level descriptive statistics, frequency response patterns, and graphical inspection of normal Q–Q plots provided some evidence for inferring univariate-normal distributional assumptions (5/7 scales). Scales “Physical Function” and “Pain Interference” exhibited more non-normality, specifically, approaching a power function due to high floor effects, which aligns with empirical findings for normal population reference values in neighboring countries (6). Scale descriptives and internal reliability estimates are displayed in Figure 1 below.

### Structural validity

**Figure 2:**
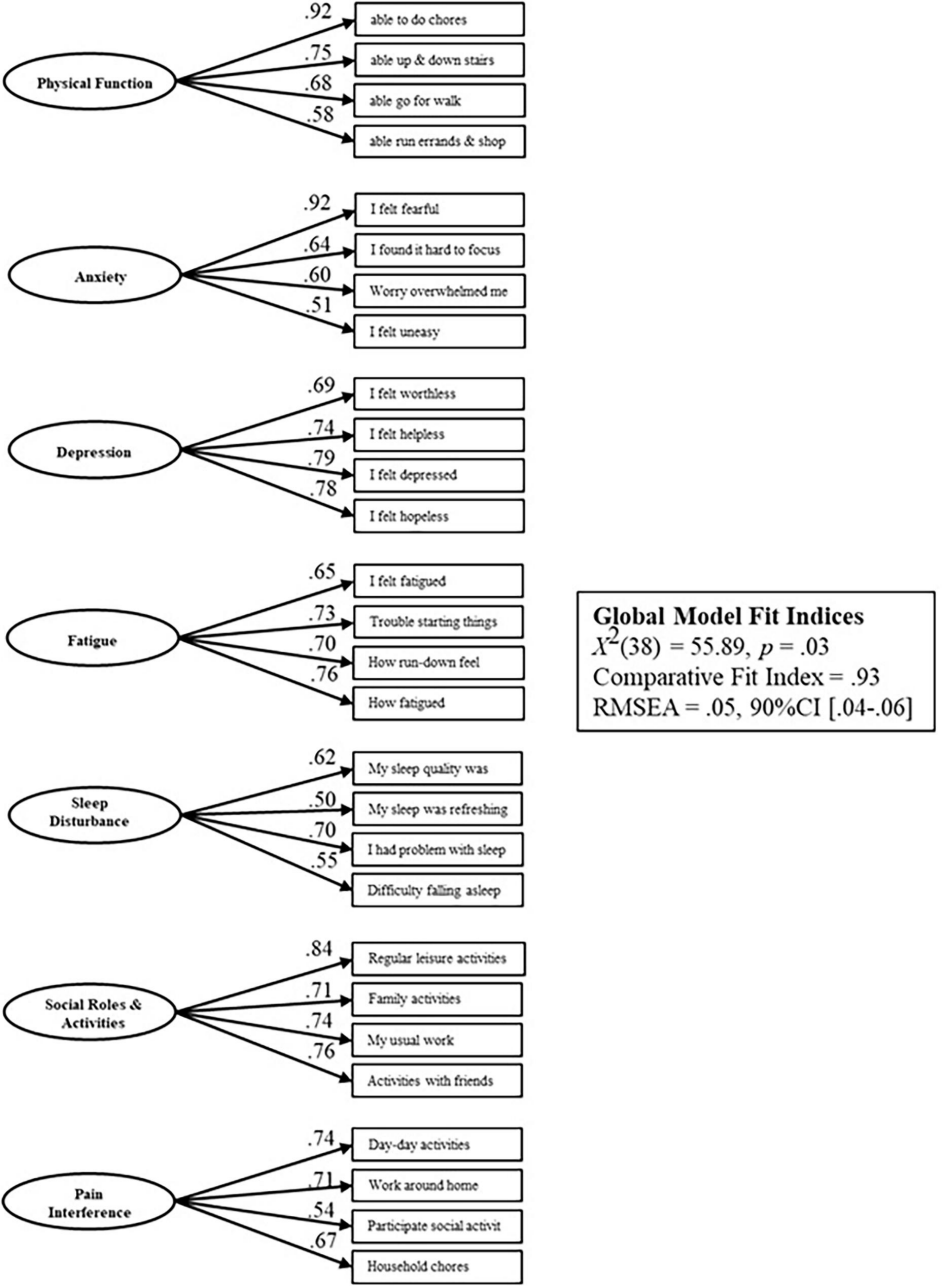
PROMIS-29 item-domain loadings and global model-fit indices.

A confirmatory factor analysis was conducted in order to inspect the structural validity of the PROMIS-29. Inspection of the item-factor loading patterns indicated significantly strong loadings in the expected direction. Furthermore, all residual item-covariances were <0.20 and RMSEA was <0.06, although the comparative fit index was slightly below the recommended 0.95 cutoff with 0.93. This is likely attributable to the relatively small sample size (*n* = 150) for such complex parameter estimations. The item-factor loadings diagram with global model fit indices is illustrated in Figure 2.

### Convergent validity

According to the COSMIN criteria, instruments measuring related or similar constructs should correlate >0.30. Inspecting the correlation matrix below (Table 2), all PROMIS-29 scales exhibited correlations >0.30 with self-rated health, with the exception of Depression, which was borderline but still significant at *r* = 0.29, *p* < 0.05. All scores also exhibited appropriately weak correlations (<0.30) with relatively unrelated variables, such as “Years in Switzerland” (discriminant validity).

**Table 2:** Intercorrelation matrix of select study variables with the PROMIS-29 scales.

Variable	Physical function	Anxiety	Depression	Fatigue	Sleep dist.	Social roles	Pain interference	Pain intensity	Age	SRH	Chronic (ρ)	Migration (ρ)	Years in CH
Physical function	1	−0.155	−0.087	−0.269**	0.186*	0.330**	−0.632**	−0.322**	−0.230**	0.581**	−0.295**	0.036	−0.171*
Anxiety	−0.155	1	0.722**	0.579**	−0.294**	−0.640**	0.105	0.043	−0.267**	−0.328**	0.017	0.187*	−0.246**
Depression	−0.087	0.722**	1	0.476**	−0.327**	−0.574**	0.057	0.003	−0.265**	−0.289**	−0.008	0.162*	−0.153
Fatigue	−0.269**	0.579**	0.476**	1	−0.548**	−0.620**	0.348**	0.195*	−0.322**	−0.527**	0.174*	0.102	−0.162
Sleep disturbance	0.186*	−0.294**	−0.327**	−0.548**	1	0.476**	−0.243**	−0.190*	0.219**	0.308**	−0.246**	−0.118	0.201*
Social roles	0.330**	−0.640**	−0.574**	−0.620**	0.476**	1	−0.364**	−0.229**	0.256**	0.414**	−0.139	−0.198*	0.179*
Pain interference	−0.632**	0.105	0.057	0.348**	−0.243**	−0.364**	1	0.538**	0.113	−0.526**	0.284**	−0.016	0.129
Pain intensity	−0.322**	0.043	0.003	0.195*	−0.190*	−0.229**	0.538**	1	0.202*	−0.239**	0.294**	−0.058	0.140
Age	−0.230**	−0.267**	−0.265**	−0.322**	0.219**	0.256**	0.113	0.202*	1	−0.078	0.260**	−0.187*	0.579**
Self-rated health	0.581**	−0.328**	−0.289**	−0.527**	0.308**	0.414**	−0.526**	−0.239**	−0.078	1	−0.419**	−0.002	−0.089
Chronic (ρ)	−0.295**	0.017	−0.008	0.174*	−0.246**	−0.139	0.284**	0.294**	0.260**	−0.419**	1	−0.066	0.100
Migration (ρ)	0.036	0.187*	0.162*	0.102	−0.118	−0.198*	−0.016	−0.058	−0.187*	−0.002	−0.066	1	−0.396**
Years in CH	−0.171*	−0.246**	−0.153	−0.162	0.201*	0.179*	0.129	0.140	0.579**	−0.089	0.100	−0.396**	1

*p < .05, **p < .01.

## Discussion

Despite the substantial prevalence of English speakers in Switzerland (6%), little is known about their health status or system use due to health surveys being administered primarily in German, French, and Italian. This study aimed to explore the feasibility of collecting English respondents, including data quality. Specifically, we informally solicited the “uptake” of English-speaking respondents via purposive sampling and examined the psychometric performance of the PROMIS-29 instrument. Findings are detailed further below.

First, regarding feasible “uptake” of English-speaking respondents, our purposive sampling in 1 month resulted in *n* = 215 respondents, of which 156 completed the survey (73% completion). This is relatively high compared to meta-analytic estimates of survey completion rates in the social and health sciences (7). Our distinct sampling approach prohibits us from commenting on potential non-response bias, but the completion rate compares to 72% for the most recent Swiss Household Panel [χ^2^_(1)_ *=* 0.10, *p* = 0.75] (8).

Second, regarding descriptives and reliability, the PROMIS-29 exhibited acceptable distributional assumptions. The higher floor effects for “Physical Function” and “Pain Interference” are consistent with findings from neighboring countries and may be explained by the administration of a patient instrument to a general population (4). Furthermore, all the scales exhibited satisfactory reliability (≥0.70) (4).

Finally, structural validity was supported, although the borderline global model fit should be inspected in a larger sample, which is currently being fielded. Furthermore, convergent validity was supported, and the network of external correlates should be expanded in future research. Despite these preliminary findings, future research should focus on omitted validity aspects, namely, measurement error and responsiveness (longitudinal), criterion validity (clinical study), and cross-cultural validity (multilingual administration). We detail additional strengths and limitations of our study below.

## Strengths and limitations

Our study has several limitations. Because of time and resource constraints, we optimized our sampling methodology by using purposive sampling in urban centers in Switzerland. Future studies, therefore, should broaden sampling frames and perhaps incentivize respondents for better representativeness. Similarly, we must note the potential for selection bias in our sample, as the respondents were not thoroughly screened as native or fluent English speakers. That is, some respondents may be Swiss or other expat nationalities who happen to speak fluent English and were interested in the study. The precision of our sample frame was not of primary concern in this feasibility-stage study. Although we inquired about chronic conditions, we failed to inquire about health services utilization, and this would be an important next step for future research to compare with the latest meta-analytic estimates (9). Furthermore, in order to minimize survey length, we included only a single-item self-rated health measure. Additional measures should be included to compare with the PROMIS-29 instrument. Finally, several COSMIN criteria remain to be examined, such as criterion validity, responsiveness, and cross-cultural validity (4). Regarding the latter, a “parent” study of this English-based pilot will provide a national representation of PROMIS-29 scores in Switzerland across German, French, and Italian regions. These scores will be examined for cross-cultural validity. Finally, although the 73% completion rate is comparable with other national health surveys in Switzerland, it must be noted that a majority of these dropouts occurred at the beginning of the survey, but did not continue after the demographics section. This could be attributable to administration error, but perhaps, more likely, it is due to the convention of providing demographic information at the end of surveys in German-speaking countries.

## Conclusion

We found a high completion rate by English-speaking respondents to a general health survey in Switzerland. Descriptives, internal consistency, structural validity, and convergent validity were mostly supported. Additional research is needed with more advanced sampling methodologies, more comprehensive questioning regarding healthcare utilization, and changes in scores over time. One potential policy implication is understanding the value-add of English surveys in expat or migrant populations compared to native Swiss languages, such as German, French, and Italian.

## Data Availability

The raw data supporting the conclusions of this article will be made available by the authors, without undue reservation.
