# Supplementary material for: A cross-sectional feasibility study of an English-language survey in Switzerland using the Patient-Reported Outcomes Measurement Information System-29
Source: Front Rehabil Sci. 2026 Jul 31;7:1840636. doi: 10.3389/fresc.2026.1840636 (PMC13473443; doi:10.3389/fresc.2026.1840636)
Supplement: Supplementary file 1 [file Supplementaryfile1.docx]

Please respond to each question or statement by marking one box per row.

| **Physical Function** | | **Without any**  **difficulty** | **With a little**  **difficulty** | **With some**  **difficulty** | **With much**  **difficulty** | **Unable to do** |
| --- | --- | --- | --- | --- | --- | --- |
| PFA11 | Are you able to do chores such as vacuuming or yard work? ......................... | 🞎  5 | 🞎  4 | 🞎  3 | 🞎  2 | 🞎  1 |
|  |  | | | | | |
| PFA21 | Are you able to go up and down stairs  at a normal pace? ...................................... | 🞎  5 | 🞎  4 | 🞎  3 | 🞎  2 | 🞎  1 |
|  |  | | | | | |
| PFA23 | Are you able to go for a walk of at least  15 minutes?............................................... | 🞎  5 | 🞎  4 | 🞎  3 | 🞎  2 | 🞎  1 |
|  |  | | | | | |
| PFA53 | Are you able to run errands and shop? ..... | 🞎  5 | 🞎  4 | 🞎  3 | 🞎  2 | 🞎  1 |
|  | **Anxiety**  **In the past 7 days…** | **Never** | **Rarely** | **Sometimes** | **Often** | **Always** |
| EDANX01 | I felt fearful ............................................... | 🞎  1 | 🞎  2 | 🞎  3 | 🞎  4 | 🞎  5 |
|  |  | | | | | |
| EDANX40 | I found it hard to focus on anything  other than my anxiety ............................... | 🞎  1 | 🞎  2 | 🞎  3 | 🞎  4 | 🞎  5 |
|  |  | | | | | |
| EDANX41 | My worries overwhelmed me ................... | 🞎  1 | 🞎  2 | 🞎  3 | 🞎  4 | 🞎  5 |
|  |  | | | | | |
| EDANX53 | I felt uneasy .............................................. | 🞎  1 | 🞎  2 | 🞎  3 | 🞎  4 | 🞎  5 |
|  | **Depression**  **In the past 7 days...** | **Never** | **Rarely** | **Sometimes** | **Often** | **Always** |
| EDDEP04 | I felt worthless .......................................... | 🞎  1 | 🞎  2 | 🞎  3 | 🞎  4 | 🞎  5 |
|  |  | | | | | |
| EDDEP06 | I felt helpless............................................. | 🞎  1 | 🞎  2 | 🞎  3 | 🞎  4 | 🞎  5 |
|  |  | | | | | |
| EDDEP29 | I felt depressed.......................................... | 🞎  1 | 🞎  2 | 🞎  3 | 🞎  4 | 🞎  5 |
|  |  | | | | | |
| EDDEP41 | I felt hopeless............................................ | 🞎  1 | 🞎  2 | 🞎  3 | 🞎  4 | 🞎  5 |
|  | **Fatigue**  **During the past 7 days…** | **Not at all** | **A little bit** | **Somewhat** | **Quite a bit** | **Very much** |
| HI7 | I feel fatigued.......................................... | 🞎  1 | 🞎  2 | 🞎  3 | 🞎  4 | 🞎  5 |
|  |  | | | | | |
| AN3 | I have trouble starting things because I am tired ................................................... | 🞎  1 | 🞎  2 | 🞎  3 | 🞎  4 | 🞎  5 |

| **Fatigue**  **In the past 7 days…** | | **Not at all** | **A little bit** | **Somewhat** | **Quite a bit** | **Very much** |
| --- | --- | --- | --- | --- | --- | --- |
| FATEXP41 | How run-down did you feel on average? ....................................... | 🞎  1 | 🞎  2 | 🞎  3 | 🞎  4 | 🞎  5 |
|  |  | | | | | |
| FATEXP40 | How fatigued were you on  average? ....................................... | 🞎  1 | 🞎  2 | 🞎  3 | 🞎  4 | 🞎  5 |
|  | **Sleep Disturbance In the past 7 days…** | **Very poor** | **Poor** | **Fair** | **Good** | **Very good** |
| Sleep109 | My sleep quality was ................... | 🞎  5 | 🞎  4 | 🞎  3 | 🞎  2 | 🞎  1 |
|  | **In the past 7 days…** | **Not at all** | **A little bit** | **Somewhat** | **Quite a bit** | **Very much** |
| Sleep116 | My sleep was refreshing. ............. | 🞎  5 | 🞎  4 | 🞎  3 | 🞎  2 | 🞎  1 |
|  |  | | | | | |
| Sleep20 | I had a problem with my sleep ..... | 🞎  1 | 🞎  2 | 🞎  3 | 🞎  4 | 🞎  5 |
|  |  | | | | | |
| Sleep44 | I had difficulty falling asleep ....... | 🞎  1 | 🞎  2 | 🞎  3 | 🞎  4 | 🞎  5 |
|  | **Ability to Participate in Social Roles and Activities** | |  |  |  |  |
|  |  | **Never** | **Rarely** | **Sometimes** | **Usually** | **Always** |
| SRPPER11_CaPS | I have trouble doing all of my  regular leisure activities with others ............................................ | 🞎  5 | 🞎  4 | 🞎  3 | 🞎  2 | 🞎  1 |
|  |  | | | | | |
| SRPPER18_CaPS | I have trouble doing all of the family activities that I want to do.................................................. | 🞎  5 | 🞎  4 | 🞎  3 | 🞎  2 | 🞎  1 |
|  |  | | | | | |
| SRPPER23_CaPS | I have trouble doing all of my usual work (include work at home) ........................................... | 🞎  5 | 🞎  4 | 🞎  3 | 🞎  2 | 🞎  1 |
|  |  | | | | | |
| SRPPER46_CaPS | I have trouble doing all of the activities with friends that I  want to do..................................... | 🞎  5 | 🞎  4 | 🞎  3 | 🞎  2 | 🞎  1 |
|  | **Pain Interference In the past 7 days…** | **Not at all** | **A little bit** | **Somewhat** | **Quite a bit** | **Very much** |
| PAININ9 | How much did pain interfere with your day to day activities? ... | 🞎  1 | 🞎  2 | 🞎  3 | 🞎  4 | 🞎  5 |
|  |  | | | | | |
| PAININ22 | How much did pain interfere with work around the home?........ | 🞎  1 | 🞎  2 | 🞎  3 | 🞎  4 | 🞎  5 |
|  |  | | | | | |
| PAININ31 | How much did pain interfere with your ability to participate  in social activities? ....................... | 🞎  1 | 🞎  2 | 🞎  3 | 🞎  4 | 🞎  5 |

| **Pain Interference**  **In the past 7 days…** | | **Not at all** | | **A little bit** | | **Somewhat** | | **Quite a bit** | | | **Very much** | |
| --- | --- | --- | --- | --- | --- | --- | --- | --- | --- | --- | --- | --- |
| PAININ34 | How much did pain interfere  with your household chores? ......... | 🞎  1 | |  | 🞎  2 |  | 🞎  3 |  | 🞎  4 |  |  | 🞎  5 |
|  | **Pain Intensity**  **In the past 7 days…** | | | | | | | | | | | |
| Global07 | How would you rate your pain  on average? .................................... | 🞎  0 | 🞎  1 | 🞎  2 | 🞎  3 | 🞎  4 | 🞎  5 | 🞎  6 | 🞎  7 | 🞎  8 | 🞎  9 | 🞎  10 |
|  |  | **No**  **pain** |  |  |  |  |  |  |  |  |  | **Worst pain**  **imaginable** |
